# GnRH-Induced Ca^2+^ Signaling Patterns and Gonadotropin Secretion in Pituitary Gonadotrophs. Functional Adaptations to Both Ordinary and Extraordinary Physiological Demands

**DOI:** 10.3389/fendo.2013.00127

**Published:** 2013-09-30

**Authors:** Maria Luisa Durán-Pastén, Tatiana Fiordelisio

**Affiliations:** ^1^Departamento de Neurociencia Cognitiva, Instituto de Fisiología Celular, Universidad Nacional Autónoma de México (UNAM), México DF, México; ^2^Departamento de Ecología y Recursos Naturales, Facultad de Ciencias, Universidad Nacional Autónoma de México (UNAM), México DF, México

**Keywords:** pituitary, gonadotrophs, calcium, gonadotropins, GnRH, secretion

## Abstract

Pituitary gonadotrophs are a small fraction of the anterior pituitary population, yet they synthesize gonadotropins: luteinizing (LH) and follicle-stimulating (FSH), essential for gametogenesis and steroidogenesis. LH is secreted via a regulated pathway while FSH release is mostly constitutive and controlled by synthesis. Although gonadotrophs fire action potentials spontaneously, the intracellular Ca^2+^ rises produced do not influence secretion, which is mainly driven by Gonadotropin-Releasing Hormone (GnRH), a decapeptide synthesized in the hypothalamus and released in a pulsatile manner into the hypophyseal portal circulation. GnRH binding to G-protein-coupled receptors triggers Ca^2+^ mobilization from InsP_3_-sensitive intracellular pools, generating the global Ca^2+^ elevations necessary for secretion. Ca^2+^ signaling responses to increasing (GnRH) vary in stereotyped fashion from subthreshold to baseline spiking (oscillatory), to biphasic (spike-oscillatory or spike-plateau). This progression varies somewhat in gonadotrophs from different species and biological preparations. Both baseline spiking and biphasic GnRH-induced Ca^2+^ signals control LH/FSH synthesis and exocytosis. Estradiol and testosterone regulate gonadotropin secretion through feedback mechanisms, while FSH synthesis and release are influenced by activin, inhibin, and follistatin. Adaptation to physiological events like the estrous cycle, involves changes in GnRH sensitivity and LH/FSH synthesis: in proestrus, estradiol feedback regulation abruptly changes from negative to positive, causing the pre-ovulatory LH surge. Similarly, when testosterone levels drop after orquiectomy the lack of negative feedback on pituitary and hypothalamus boosts both GnRH and LH secretion, gonadotrophs GnRH sensitivity increases, and Ca^2+^ signaling patterns change. In addition, gonadotrophs proliferate and grow. These plastic changes denote a more vigorous functional adaptation in response to an extraordinary functional demand.

## Gonadotrophs Function and Characteristics

The reproductive function and sexual maturation is under the control of the hypothalamic-pituitary-gonadal axis. Pituitary gonadotrophs, which constitute 7–15% of the anterior pituitary gland secrete two dimeric glycoproteins, gonadotropins, luteinizing (LH) and follicle-stimulating (FSH) hormones that play an essential role in the control of steroidogenesis, gametogenesis, and ovulation (1). The regulation of their synthesis and secretion are under control of hypothalamic stimulation (gonadotropin-releasing hormone; GnRH), gonadal sex steroids (estradiol, progesterone, testosterone) and peptides (inhibins), and paracrine factors (inhibins, activins, and follistatin). The pituitary gland must adapt to different physiological changes from prepubertal to mature sexual life, therefore gonadotrophs plasticity and gonadotropins secretion are essential to produce the changes needed in different situations, for example the rapid daily hormonal variations along the reproductive female cycle. Integration of the different regulatory signals by the gonadotrophs results in the coordinated control of synthesis, packaging, and differential secretion of gonadotropins to accurately respond and control sexual maturation and normal reproductive function.

Immunocytochemical studies have demonstrated the presence of bihormonal (70%) and monohormonal (15%) gonadotrophs whose percentage shifts under different physiological conditions, such as castration or estrous cycle (2). LH and FSH have a common alpha (α) and distinct beta (β) subunit. After its synthesis in the endoplasmic reticulum (ER) and its passage trough the Golgi apparatus, hormones are delivered to the plasma membrane trough a constitutively or regulated secretory pathway; in the latter, fusion of secretory vesicles to the plasma membrane is arrested waiting for specific signals to be secreted. Gonadotropin synthesis and secretion diverges under a range of physiological and experimental conditions (3), indicating that GnRH and other regulators of gonadotropins selectively activate this pathways.

Exocytosis in excitable cells is a process highly dependent of intracellular calcium concentration ([Ca^2+^]_i_) rise, gonadotrophs as other pituitary endocrine cells display spontaneous intracellular Ca^2+^ transients in dependence of changes in the membrane electrical activity. However, this membrane potential oscillations are small and do not produce the necessary [Ca^2+^]_i_ increase to generate hormonal secretion (4, 5), as a result, basal secretion is low and not affected by extracellular Ca^2+^ (4, 6). In both cases, the principal regulation is done by GnRH, a decapeptide that is synthesized in the hypothalamus, stored in axon terminals in the median eminence, and released in a pulsatile manner into the hypophyseal portal circulation (7). Numerous studies have shown that isolated gonadotrophs in primary culture (and more recently, also gonadotrophs *in situ*) present robust and stereotyped dose-dependent intracellular Ca^2+^ signals in response to suprathreshold concentrations of GnRH (8–11), the rise produced in cytosolic [Ca^2+^] triggers gonadotropins exocytosis and synthesis.

Understanding the origin and meaning of these intracellular Ca^2+^ signals are essential to the knowledge of the physiology of normal reproduction, as well as reproductive function disorders. This review outlines different regulators of the gonadotrophs biology with special regard in the recent progress on GnRH-induced Ca^2+^ signaling and secretion in pituitary gonadotrophs, both at the cellular and tissue level.

## Ca^2+^ Signals Induced by GnRH and Other Secretagogues

**Figure 1:**
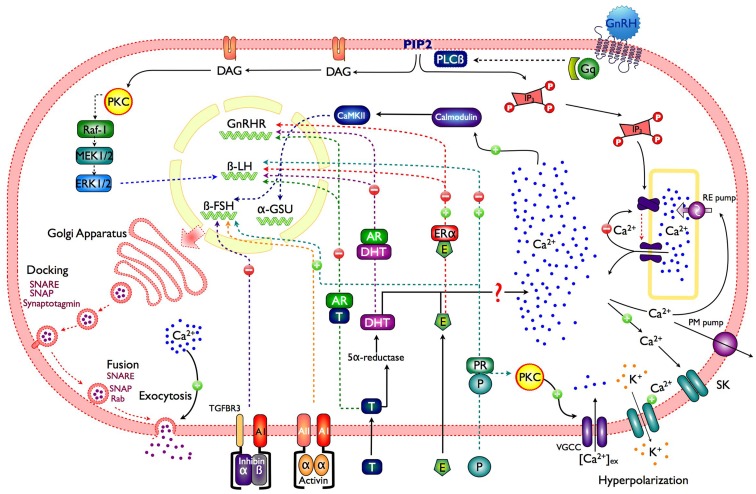
**Schematic representation of a gonadotroph illustrating the main control pathways of gonadotropin synthesis and secretion**. GnRH, gonadotropin-releasing hormone; Gq, protein Gq/_11_; PLCβ, phospholipase C; PIP2, phosphatidylinositol 4,5 bisphosphate; DAG, diacylglycerol; IP_3_, inositol 1,4,5-trisphosphate; PKC, protein kinase C; VGCC, voltage-gated calcium channels; CaMKII, calcium calmodulin type II kinase; RE pump, endoplasmic reticulum Ca^2+^ pump; PM pump, plasma membrane Ca^2+^ pump; SK, small conductance calcium-activated potassium channels; P, progesterone; PR, progesterone receptor; E, estradiol; ERα, estrogen receptor α; T, testosterone; AR, androgen receptor; Raf, serine/threonine kinase; MEK, mitogen-activated protein kinase; ERK, extracellular-signal-regulated kinases.

In order to mediate multiple effects such as secretion, synthesis, and phenotype maintenance, the GnRH variants in different species interact with their receptor (GnRHR), which is a member of the rhodopsin-like G-protein-coupled receptors (GPCR) superfamily (12). Upon GnRH binding to the GnRHRs in the gonadotroph membrane, the α subunit of the Gq/_11_ protein dissociates and activates phospholipase C (PLC-β), resulting in the rapid hydrolysis of phosphatidylinositol 4, 5-biphosphate (PIP2) and the production of two second messengers: diacylglycerol (DAG) and inositol 1,4,5-trisphosphate (InsP_3_); long lasting GnRH stimulation (∼5–10 min) could also activate phospholipase D (PLD) and phospholipase A_2_ (PLA_2_) (12). InsP_3_ generates Ca^2+^ mobilization from intracellular pools, and DAG triggers protein kinase C (PKC) activation which in turn reduces depolarization-mediated Ca^2+^ influx, while increasing gonadotropin secretion (13) (Figure 1). PKC sensitizes the secretory machinery to Ca^2+^ (14), which explain why GnRH application is more effective to induce secretion than membrane depolarization or caged Ca^2+^ photolysis (5). PKC activation is also involved in other exocytosis-associated processes, like GnRH self-priming and cytoskeletal rearrangement (3).

In the lumen of the ER, [Ca^2+^] is maintained higher (between 10 and 250 μM free) than in the cytosol (50–250 nM) by the pumping activity of the sarco-ER Ca^2+^ ATPase (SERCA) located in the ER membrane (15). This membrane holds intracellular channel that allow Ca^2+^ efflux from the ER down its concentration gradient; the InsP_3_ receptor (InsP_3_R), a ligand-gated Ca^2+^ channel that opens after InsP_3_ binding (16). Besides InsP_3_ binding, Ca^2+^ interaction with high-affinity (activation) sites on the cytoplasmic side of the InsP_3_R is essential for channel opening. In fact, Ca^2+^ and InsP_3_ operate as co-agonists. Ca^2+^ signal amplification and spreading phenomena, involving assemblies of InsP_3_Rs originate from this synergistic role of Ca^2+^ (17). The large and abrupt [Ca^2+^]_i_ increase, triggered by InsP_3_Rs activation results from the combination of Ca^2+^ released and its amplification by Ca^2+^ induced Ca^2+^ release [CICR; (18)]. Even if cytosolic InsP_3_ remains high, Ca^2+^ efflux often ceases because Ca^2+^ binds to a low-affinity (inactivating) site of the receptor, which closes the InsP_3_R channel. This occurs when cytosolic Ca^2+^ close to the InsP_3_Rs is high, i.e., after an episode of fast release. As in most pituitary cells, agonist stimulation in gonadotrophs produce a [Ca^2+^]_i_ peak which decays to sustained Ca^2+^ level (plateau phase). At intermediate GnRH concentration the initial Ca^2+^ spike is often followed by large [Ca^2+^]_i_ oscillations resulting from opening and closing cycles of the InsP_3_R channels as a consequence of [Ca^2+^]_i_ fluctuations near its cytoplasmic side (19). The frequency of these Ca^2+^ oscillations is determined by the dose of GnRH applied and the intracellular (InsP_3_) reached (20) (Figure 1).

Gonadotropin-releasing hormone-induced [Ca^2+^]_i_ oscillations can be reproduced with mathematical models that include a Ca^2+^ gradient between the ER lumen and the cytosol maintained by a SERCA Ca^2+^ pump, Ca^2+^ influx trough voltage-gated Ca^2+^ channels, and InsP_3_R channels co-activated by InsP_3_ and low [Ca^2+^]_i_, and inactivated by high [Ca^2+^]_i_ (8, 15, 21, 22). Nonetheless, Ca^2+^ oscillations in real cells requires the precise coordination of Ca^2+^ mobilization/uptake/extrusion mechanisms, it is for it that immortalized gonadotroph cell lines αT3–1 (21) and LβT2 (23) are not good cell models for studies on GnRH-induced calcium signaling and modulation of voltage-gated calcium influx, as well as goldfish (24, 25) and immature mammalian gonadotrophs, since these cells respond to GnRH with non-oscillatory amplitude-modulated Ca^2+^ signals. When SERCA pumps in gonadotrophs are blocked by thapsigargin, the agonist-induced Ca^2+^ oscillations become non-oscillatory biphasic responses (8, 26). Therefore different factors, i.e., the amount and speed of InsP_3_ production, the total number of InsP_3_R channels available for activation, the rate of Ca^2+^ leakage from the store and the efficiency of the SERCA Ca^2+^ pump vary from cell to cell, and they ultimately determine the characteristics of gonadotrophs Ca^2+^ signaling patterns. It is important to note that the oscillatory behavior is intrinsic to the Ca^2+^ handling properties of gonadotrophs (17).

Gonadotropin-releasing hormone produces Ca^2+^ oscillations: i.e., large Ca^2+^ spikes, arising from a flat baseline as well as smaller sinusoidal Ca^2+^ oscillations superimposed on an elevated plateau. Under sustained GnRH stimulation, the amplitude of these Ca^2+^ spikes gradually diminishes, probably due to intracellular Ca^2+^ pool depletion, until a “plateau” without oscillations is reached. Ca^2+^ influx through voltage-gated Ca^2+^ channels is essential to maintain this plateau, and also for the replenishment of intracellular Ca^2+^ pools. GnRH induces continuous AP firing periodically interrupted by hyperpolarizations, which occur in phase with each Ca^2+^ elevation, and resulting from the opening of Ca^2+^ dependent SK-type K^+^ channels (6, 27). Immediately after each hyperpolarization, the cell fires a burst of APs, which open Ca^2+^ channels allowing Ca^2+^ influx, predominantly high-voltage – activated L-type calcium channels. This Ca^2+^ entry does not contribute to Ca^2+^ elevation or gonadotropin secretion, but is crucial for refilling the intracellular Ca^2+^ pools (20) (Figure 1).

Oscillatory Ca^2+^ signals in gonadotrophs can also be elicited by endothelin (ET) (28, 29), pituitary adenylate cyclase-activating polypeptide, (PACAP) (30), and substance P (SP) (31). Conversely, neuropeptide Y (NPY) and melatonin, in neonatal gonadotrophs, inhibit GnRH-induced Ca^2+^ signals and gonadotropin secretion. Lactotrophs, gonadotrophs, and somatotrophs produce ETs, and gonadotrophs express ET receptors (32) under the control of ovarian steroid hormones, suggesting a paracrine function. ET binding, leads to Gq/_11_ activation, intracellular Ca^2+^ fluctuations, and gonadotropin secretion (29). SP, which is a weaker agonist than GnRH, produces amplitude-modulated [Ca^2+^]_i_ responses and secretion in gonadotrophs (31), being the first phase of secretion dependent of intracellular Ca^2+^ release, and the second phase Ca^2+^ influx-dependent. The hypothalamic factor PACAP which stimulates cAMP production and potentiates gonadotropin release (33), also induces Ca^2+^ oscillations in rat gonadotrophs through activation of PVR1, a G-protein-coupled receptor and InsP_3_ production (30). The activation of coupled G_i/o_ melatonin receptors MT1 and MT2, expressed in gonadotrophs only at neonatal stage, inhibits both calcium influx through voltage-gated calcium channels and calcium mobilization from intracellular stores, decreasing intracellular cAMP production and protein kinase A (PKA) activity, with a consequent diminution on gonadotropin secretion (34–36); tonic melatonin inhibition of immature gonadotrophs prevents premature initiation of puberty. NPY inhibits GnRH-induced Ca^2+^ signaling and LH release (37); its receptors Y1 and Y5 expression on gonadotrophs is regulated by estrogens (38).

## Ca^2+^ Signaling Patterns and Secretion in Gonadotrophs is Dependent on GnRH Concentration

Dissociated pituitary gonadotrophs respond to increasing doses of GnRH with a stereotyped progression of intracellular Ca^2+^ signaling: i.e., subthreshold GnRH concentrations produce either small monophasic Ca^2+^ transients or irregular, small Ca^2+^ spikes. With higher GnRH concentrations (0.1–10 nM) regular, oscillatory, frequency-modulated, large Ca^2+^ transients (baseline Ca^2+^ spiking) are produced. Eventually (∼50–100 nM GnRH), these Ca^2+^ spikes fuse into an amplitude-modulated biphasic Ca^2+^ response (9, 10, 39) which comprises two variants; biphasic oscillatory and biphasic non-oscillatory, also known as spike-plateau (40). It is reasonable to assume that different Ca^2+^ release patterns observed with increasing doses of GnRH underlie the dose-dependent increase of gonadotropin secretion. Nonetheless, it has also been suggested that these patterns encode other cell functions. For instance, spike-plateau Ca^2+^ responses were associated to LH secretion and oscillatory Ca^2+^ responses to the synthesis of LH β-subunits (9). Later, it was established that GnRH-induced Ca^2+^ oscillations trigger exocytosis (41) and that both oscillatory and spike-plateau Ca^2+^ signals can initiate LH release (10, 40). Furthermore, gonadotrophs do not respond in the same way to the secretagogue: i.e., individual cells can respond with different patterns of activity to the same GnRH concentration (40). Conversely, when the same dose of GnRH is applied repetitively, individual cells respond with similar latency and signaling pattern (11). It remains to be established which cellular aspects determine the Ca^2+^ signals displayed by individual gonadotrophs in response to GnRH and how these different patterns affect gonadotropin synthesis and secretion. Moreover, LH and FSH are secreted through parallel pathways (see below) and hormones that alter their synthesis, release, and/or storage can dynamically regulate their output.

## Gonadotropin Exocytosis. Contribution of VGCC-Mediated Ca^2+^ Influx and Intracellular Ca^2+^ Release

A rise in [Ca^2+^]_i_ is the key signal to trigger regulated exocytosis in neuronal and endocrine tissues. Endocrine cell models used to study the role of Ca^2+^ in exocytosis include adrenal chromaffin and PC12 cells (42–46), pancreatic β cells (47–49), and pituitary cells (6, 50). Cytosolic Ca^2+^ levels regulate several maturation steps that secretory vesicles must undergo prior to fusion, like priming of secretory vesicles (51). An entirely different phenomena occurs when [Ca^2+^]_i_ rises abruptly, promoting the fusion of docked secretory vesicles with the plasma membrane (47, 52). In contrast to nerve synapses, where Ca^2+^ influx is primarily responsible for this abrupt [Ca^2+^]_i_ rise, exocytosis in endocrine cells is triggered to a large extent by Ca^2+^ released from intracellular stores (17, 53).

Ca^2+^ controls the fusion of secretory vesicles with the plasma membrane to release neurotransmitters and hormones when is needed [regulated exocytosis, (51)]. The first phase of GnRH-induced exocytosis in gonadotrophs is mediated by InsP_3_-sensitive Ca^2+^ pools, while the second “plateau” phase of secretion involves voltage-gated Ca^2+^ influx (54). GnRH-InsP_3_ induced Ca^2+^ oscillations produce much greater exocytosis than the simple general rise in [Ca^2+^]_i_ induced by micropipette injection or uncaging [Ca^2+^]_i_ (5, 41). This suggests that in contrast with other pituitary cell types, the formation of sub-plasmalemmal microdomains of high Ca^2+^ in gonadotrophs is insufficient to induce vesicular fusion. Instead large Ca^2+^ signals that propagate across the entire cell are needed to accomplish this task (6). Exocytosis can be directly monitored electrically as changes in membrane capacitance due to the addition of new plasma membrane. Using capacitance measurements, exocytosis is detected in gonadotrophs whenever [Ca^2+^] rises above 300 nM (55), but for strong exocytosis high [Ca^2+^]_i_ with half maximal concentration of 16 μM are required (6). When the responses induced by GnRH are oscillatory, step increases in membrane capacitance can be seen in each Ca^2+^ spike (40, 41, 55). The first Ca^2+^ oscillations elicit the largest exocytosis events, returning to full capacity within about 2 min (5). GnRH-induced secretion continues in the absence of external Ca^2+^, but ceases when [Ca^2+^] rises are blocked by the introduction of a strong intracellular Ca^2+^ buffer (41).

Secretory granules must undergo a well-defined series of events: (1) recruitment, (2) tethering at the plasma membrane, (3) priming, and (4) vesicle fusion with the plasma membrane. Regulated hormone secretion is a Ca^2+^-dependent exocytosis that uses the secretory vesicle synaptotagmin as the Ca^2+^ sensor and is mediated by SNARE (soluble *N*-ethylmaleimide-sensitive factor attachment protein receptors) proteins as effectors. Syntaxin, SNAP25 (synaptosome-associated protein of 25 kDa in molecular weight), and synaptobrevin (vesicle-associated membrane protein, VAMP, also termed vSNARE) constitute SNARE proteins. Syntaxin and SNAP25 (also known as “target” tSNAREs) are the plasma membrane proteins to which VAMP couples (Figure 1). Then, vSNAREs and tSNAREs form trans-SNARE complexes, which join secretory vesicles and plasma membrane (56–58). Vesicle priming, another Ca^2+^-dependent step in exocytosis probably involves early SNARE complex formation (particularly tSNARE), before its association to the trans-SNAREs. Finally, synaptotagmin detects the [Ca^2+^] elevation and provides the extra drive needed to overcome the energy barrier of lipid-to-lipid interaction, allowing membrane fusion (58). The use of high-resolution microscopy techniques have allowed to demonstrate in PC12 cells that tSNARE molecules are distributed on the plasma membrane in areas of low and high density, and in contrast to current models of SNARE-driven membrane fusion (59), this data suggest that secretory vesicles are targeted over areas of low tSNARE density as sites of docking, hence a relatively low number of tSNAREs close to the secretory vesicle (less than seven) are sufficient to drive membrane fusion. Moreover, using atomic forces microscopy and scanning electron microscopy it has been described that gonadotrophs mainly present “single and simple fusion pore” with diameter ranging from 100 to 500 nm, which appear more frequently after stimulation with GnRH; this pore configuration supports the idea of a “kiss and stay” mechanism for the exocytosis process (60), in addition pores of 20–40 nm diameter have also been found, probably representing the constitutive pathway of gonadotropins (60).

## FSH and LH Differential Secretion Under Physiological Conditions

Along the follicular phase of the estrus cycle, LH secretion is maximal while FSH secretion is reduced; even though gonadotrophs secrete both hormones, the mechanisms underlying this differential release are unclear. FSH appears to be released mostly through the constitutive pathway in accordance to its rate of synthesis. Conversely, LH-containing granules are released through the regulated pathway in response to GnRH, with no effect on LHβ mRNA production (61). Moreover, LH and FSH appear to be packaged into different secretory granules (62). Large, moderately electron-dense granules show antigenicity for FSH, LH, and chromogranin A (CgA), while smaller, electron-dense storage granules released by GnRH contain LH and secretogranin II (SgII) (3); thereby protein sorting domains in the β subunit of gonadotropins and the association with certain proteins may be responsible for differential sorting and packaging of LH and FSH into different secretory granules (3). The movement of these granules toward the membrane defining a secretory pathway and differential exocytosis could explain the disparity on the gonadotropins secretion (63). Accordingly, in LβT2 mouse cells, FSH released in response to activin/GnRH is constitutively secreted via a granin-independent pathway; while LH is released in response to GnRH is co-released with SgII via a regulated, granin-dependent pathway (64).

Gonadotropin subunits (α-GSU, FSHβ, and LHβ) mRNAs levels, which reflect changes in gene transcription in pituitary gonadotrophs, are GnRH pulse frequency modulated (65–67). GnRH pulses (30–60 min interval), preferentially increases synthesis and secretion of LH by the mediation of the transcription factor Egr-1 (68–71); whereas slower GnRH pulsing (120–240 min interval) favors FSH secretion (65–67, 72) by the activation of PKA (73–75). There is no a definitive explanation to how GnRH pulses can activate in a different manner gonadotropin subunit gene transcription; nevertheless several routes have been proposed which may contribute to this regulation; one is through the increase on Ca^2+^ levels and PKC activation, which as a consequence activated mitogen-activated protein kinase (MAPK) cascade, culminating in an activation of extracellular-signal-regulated kinase (ERK) 1/2, cJun NH2-terminale kinase (JNK), p38 MAPK, and ERK 5 (76–80), it is also believe that the rise in [Ca^2+^]_i_, activates a calcium/calmodulin-dependent kinase II (CAMK2), whose autophosphorylation could be important in transmitting Ca^2+^ pulse frequency and amplitude signals, as fast and high-amplitude Ca^2+^ influxes, which results in greater and/or sustained Ca^2+^/CALM1 levels (79, 81) (Figure 1). GnRH pulses at lower frequency selectively increase the expression of PACAP and its receptor (PAC1-R) in gonadotrophs (82), where they subsequently stimulate the synthesis of gonadotropin subunits (83).

**Figure 2:**
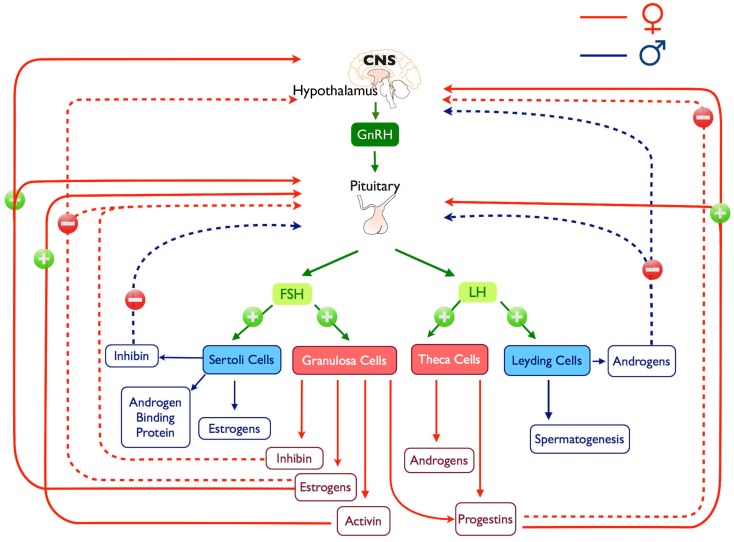
**Representation of the hypothalamus-pituitary-gonadal axis, positive and negatives feedback loops and products are illustrated**.

Gonadotropin-releasing hormone-induced LH and FSH synthesis and secretion are modulated by steroid hormones, such as estrogen, progesterone, and testosterone, in addition to peptide hormones, such as activin, inhibin, and follistatin (Figures 1 and 2). This modulation occurs principally through gonadal feedback at the pituitary and hypothalamus level (84–86). During most part of the female reproductive cycle and in males, pulsatile GnRH release drives tonic gonadotropin secretion (84, 87) while steroids and inhibins provide negative feedback to limit further gonadotropin stimulation and maintaining low circulating levels of gonadotropins; in females, this happens until the pre-ovulatory surge when, in response to low levels of progesterone (88) and an increase in estrogen, feedback switches to positive (89), producing changes on GnRHergic neurons (90) and gonadotrophs (91), which results in increased LH and FSH secretion (Figure 2). In some female species a secondary FSH surge occurs after ovulation when LH levels are already low, this rise produces the recruitment of the next cohort of follicles and it is GnRH independent (92) and more likely depends on the reduction of circulating inhibin (93).

Estradiol (E) exerts a direct action at the pituitary level through its α-receptor (94–97), increasing gonadotroph responsiveness to GnRH (98–100) raising synthesis and insertion of GnRH receptor into gonadotroph membrane (86, 91, 101–104) and decreasing the concentration of GnRH needed to produce the threshold response and frequency of Ca^2+^ spiking (101, 105, 106). Nevertheless, these actions seems to be and indirect action that depends of the increased expression produced by GnRH on its own receptor (101, 103, 107–109). Besides these changes, during the gonadotropin surge, the pituitary gland shows cellular modifications, implying an augmentation on the number of secreting gonadotrophs (98, 104) and hypertrophy and re-organization of its intracellular organelles (110–112). However, it has been documented that E can act to suppress the transcriptional rate of LH subunit genes. Controversial results have been reported for FSHβ synthesis (113–117), although serum levels of both hormones increased markedly.

Progesterone (P) exerts some of its effects at hypothalamic level, decreasing GnRH secretion and pulse frequency (91, 103) contributing to the abrupt decline in gonadotropin levels. P does not inhibit LH secretion induced by GnRH (100, 118) but it can stimulate murine FSHβ promoter activity alone or in synergy with activins (103). In dependence with the time of exposition, P can either inhibit or facilitate the estrogen-induced LH surge during the rat estrous cycle (100, 103, 119). P modulates the E effect on GnRH production of LH surge by the modulation of Ca^2+^ mobilization and Ca^2+^ entry to gonadotrophs. In E-primed cells P alters the intracellular Ca^2+^ signaling patterns produced by GnRH. In the short-term P treatment shifts subthreshold [Ca^2+^]_i_ responses to oscillatory, and oscillatory to biphasic responses; in contrast, long-term P exposure led to decreased GnRH sensitivity, changing oscillatory response into subthreshold [Ca^2+^]_i_ response profiles (105, 106).

Androgens [testosterone (T) and 5α-dihydrotestosterone (DHT)] are important component of the male gonadal feedback and they act either at the hypothalamic level by regulating the secretion of GnRH into the hypophyseal portal circulation (120–122), directly at the pituitary level (99) or by the combination of both sites (123) (Figure 2).

At hypothalamic level, T reduce GnRH synthesis (122, 124–127) and pulsatile patterns of GnRH release (128–131). At pituitary level, it is known that testosterone and more dramatically DHT inhibits LH synthesis and GnRH-induced LH secretion in a concentration and time dependent manner (132–137), but increase basal FSH secretion and synthesis (138, 139). In castrated rats it has been shown that LH secretion increase (140, 141) as well as gonadotrophs size and number (140, 142, 143). These hypertrophied cells are called castration cells (144–146), and they present a dilated rough ER and an extended Golgi complex (147, 148). On these cells, the secretion granules content are progressively diminished (149) and their cisternae fused to form large vacuoles that originated the typical “signet ring cell” (148, 150, 151).

It is widely accepted that in gonadotrophs an increase in [Ca^2+^]_i_ is essential for the transduction of GnRH signal; T but specially DHT regulate GnRH-induced [Ca^2+^]_i_ variations (152) changing the type of calcium patterns (153), these effects are not seen in all species (145) and it could be related with the influence of this hormone on the regulation of the GnRH receptor density (154–156) and the change in their sensitivity to the GnRH stimulus (134).

Tobin and collaborators (153) demonstrated that in cultured gonadotrophs of gonadectomized male rats, the relationship between GnRH concentration and the type of intracellular Ca^2+^ response is altered, most gonadotrophs (∼70%) show oscillatory responses regardless of the GnRH concentration. Correlated with this results it has been demonstrated that in T or DHT treated cells, there is an inhibition of the GnRH increase in [Ca^2+^]_i_; at low GnRH doses (0.1 nM) 30% of gonadotrophs were unable to initiated threshold spiking and in the residual cells the frequency of oscillations decreased, as in controls, androgen treated cells, respond with a spike-plateau type of signal to 1 nM GnRH, but the frequency of spiking was also reduced (134, 152). Finally at high dose GnRH (100 nM) induce biphasic elevations of [Ca^2+^]_i_ with a minor reduction in the amplitude (134). Testosterone inhibits both phases of GnRH-stimulated LH secretory responses, the early extracellular Ca^2+^-independent spike phase and the sustained Ca^2+^ and extracellular Ca^2+^-dependent plateau phase (134). These results suggest that androgens act on the efficacy of the agonist to release Ca^2+^, leading to a decrease in the secretory output.

As it has been previously established, secretion of FSH and LH are not co-ordinately regulated, their discordant regulation must be related to differential intracellular responses to several stimuli, factors as activins, inhibins, and follistatin, may play a key role on establishing such differences. In this regard, activins which are produced in a variety of tissues, including gonadotrophs, stimulates FSHβ transcription (132, 157–159) and enhance its sensitivity to GnRH by up-regulation of the GnRH receptor expression (92, 160). Contrary, inhibins which are produced in Sertoli and granulosa cells as well as in gonadotrophs (161), have been shown to rapidly reduce FSHβ synthesis and secretion independently of GnRH (162), by binding to activin receptors on gonadotrophs preventing the assembly of active signaling complexes (92).

Follistatins, which are glycoprotein ubiquitously expressed (including gonadotrophs and follicle stellated cells) bind to activins with high-affinity modulating its actions (92, 132, 160, 163). Activin and follistatin function in a reciprocal feedback loop altering their secretion, internalization, and degradation (92, 114, 160, 163), modifying the rise and fall of biosynthesis and secretion across the reproductive cycle (160, 163).

One mechanism that contributes to differential FSH and LH production may be related to the observation that different patterns of GnRH pulses produce differential effects on inhibin/activin and follistatin mRNA levels (160). Estrogen, progesterone, testosterone, inhibin, activin, follistatin, and hypothalamic GnRH, may combine to distinct regulate LH and FSH during the reproductive cycle (97).

## Gonadotrophs Activity at the Tissue Level

Endocrine cells are organized in three-dimensional networks, which facilitate the coordination of the activity of thousands of individual cells to respond to different regulation factors and achieve hormone output (164, 165). The magnitude of the hormone pulses into the systemic circulation is apparently not just the simple addition of the individual endocrine activity, instead, biophysical and biochemical interactions in the whole tissue must be essential for *in vivo* organization. However, as it has been described in this and other works, most of the studies have been done in individual cell activity where this networks and relations are disrupted, due to methodological difficulties, just few recently approaches has been done in the understanding of the endocrine activity in a tissue context.

In this regard, the distribution of gonadotrophs in fixed and live slices at different female reproductive stages has been analyzed (166). Across different physiological stages, pituitary gonadotrophs shows changes in their distribution within the gland and in response to GnRH stimulation (166), this might represent and adaptation to better respond at different conditions.

**Figure 3:**
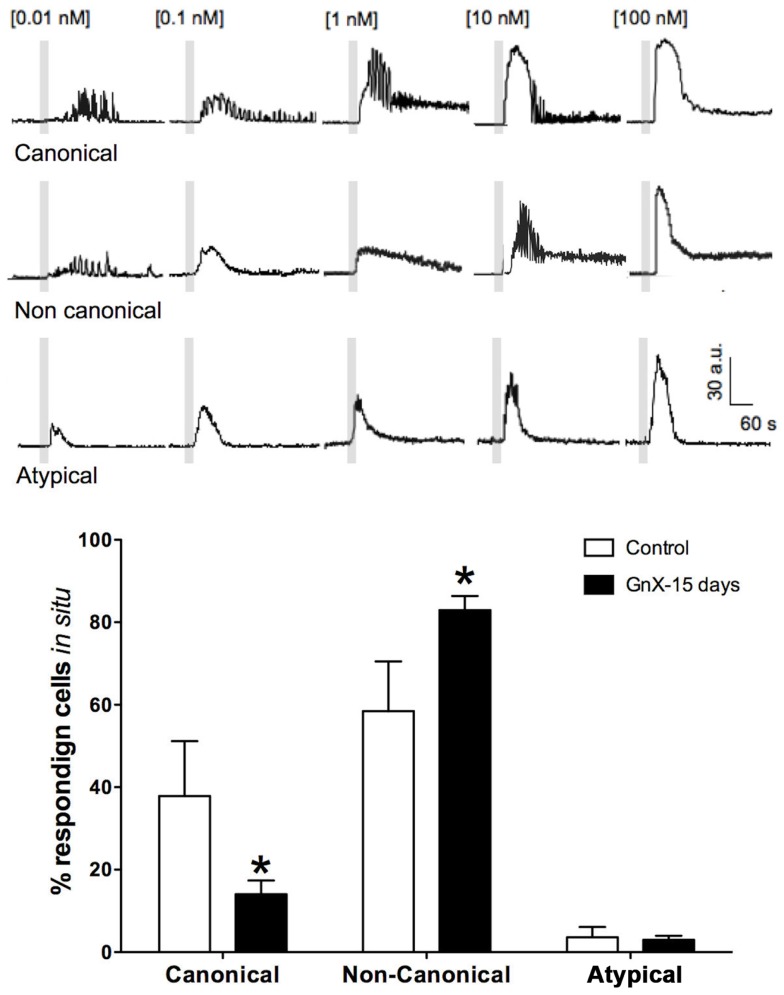
**Percentage of gonadotrophs that display different GnRH dose-response intracellular Ca^2+^ signaling patterns rises in response to increasing GnRH: canonical ([Ca^2+^]_i_ oscillations of increasing frequency at low-medium GnRH concentration and spike-plateau at saturating GnRH concentration), non-canonical (disordered sequence of oscillatory and spike-plateau [Ca^2+^]_i_ signals in response to increasing GnRH concentrations), and atypical (non-oscillatory, transient [Ca^2+^]_i_); open bars represent data from intact mice and black bars those of castrated mice after 15 post-GnX**. After orchidectomy, non-canonical responses increased, while the fraction of cells with canonical responses declined. Differences between intact and post-GnX, for both canonical and non-canonical responses are significant. *p < 0.05 versus the control (two way ANOVA with Bonferroni *post hoc* test). Parts of this figure were originally published in Durán-Pastén et al., (145).

The possibility of changes in gonadotrophs activity within its tissue context and physiological conditions was recently addressed using Ca^2+^ imaging in male mouse acute pituitary slices (11, 145). Cells in this preparation are amenable to functional studies in their native environment. We showed that rather than a constant number of gonadotrophs responding to GnRH stimulus, the number of responding cells grew with increasing GnRH concentration (GnRH), and in general, gonadotrophs Ca^2+^ signaling resembled that recorded in primary cultures (11, 145). However, Ca^2+^ imaging in acute mouse pituitary slices revealed Ca^2+^ signaling patterns unique to *in situ* conditions, gonadotrophs (58%) under increasing doses of GnRH stimulation exhibited a progression of Ca^2+^ signaling patterns termed “non-canonical” [i.e., oscillatory responses at a given (GnRH) and transient responses at both lower and higher concentrations as described before in this review; Figure 3], and some of them (3.6%) even showed atypical (non-oscillatory) responses, regardless of the (GnRH) used (145). Furthermore, responses to a given dose of GnRH varied considerably from one cell to another, reflecting a range of dose-response properties in the *in situ* gonadotroph population.

**Figure 4:**
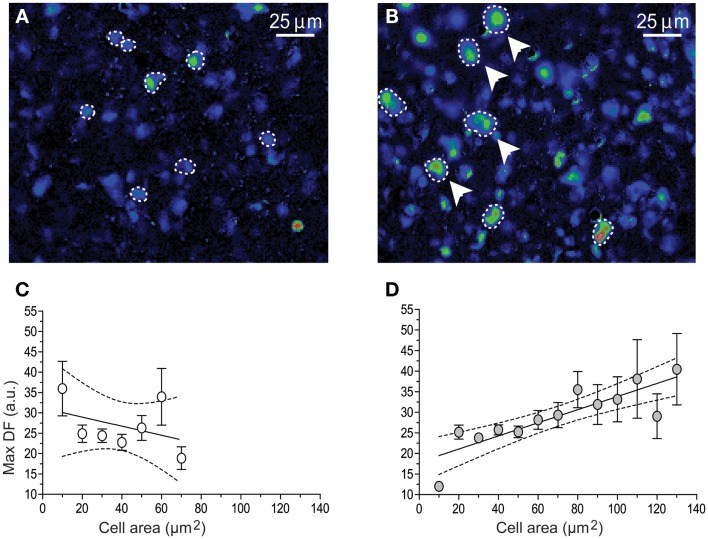
**Graphs illustrating the relation between gonadotrophs area size versus the peak amplitude of GnRH-induced Ca^2+^ transients**. Fluo-4 fluorescence images of 100 nM GnRH responding gonadotrophs (dashed lines) in intact **(A)** and 15 days post-GnX **(B)** mice pituitary slice, arrows pointed bigger gonadotrophs. **(C,D)** shows the relationship between [Ca^2+^]_i_ transients peak amplitude (Max DF) and cell area (Mean ± SE). **(C)** Intact (*n* = 6) and **(D)** 15 days post-GnX (*n* = 6) mice pituitary slices are represented; dashed line represent the confidence interval. **(C)** *y* = −0.11* ± 0.11x + 31.1 ± 5.1, *R*_2_ = 0.15, *p* > 0.05, Pearson *r* = 0.38, *p* > 0.05 and **(D)** *y* = 0.16* ± 0.02x + 17.88 ± 2.3, *R*_2_ = 0.73, *p* < 0.05, Pearson *r* = 0.85, *p* < 0.05. Parts of this figure were originally published in Durán-Pastén et al., (145).

As it has been described in this review, following the removal of the gonads, the population of pituitary gonadotrophs undergoes drastic functional and morphological modifications concomitantly with the large (five to sixfold) increase in gonadotropin secretion that characterizes this condition (123, 154, 167, 168) some changes as amplitude and frequency of GnRH-induced Ca^2+^ signaling has been reported in dissociated cells (10) and there is no difference with respect of what it has been reported in acute pituitary slices from 15 and 45 days castrated male mice (GnX) (145). Nevertheless, other characteristics on the intracellular Ca^2+^ signaling appear to be different; gonadotrophs of pituitary slices from GnX responding with “non-canonical” sequences of Ca^2+^ signaling (described earlier in this review) to increasing GnRH were significantly augmented (80% of GnRH responding gonadotrophs) and “canonical” sequences were significantly reduced (145) (Figure 3), indicating that probably this sequences of Ca^2+^ signaling in response to GnRH are modulated by paracrine and systemic factors as testosterone, allowing gonadotrophs to adapt to different physiological requirements. Additionally, median effective dose (ED50) for GnRH decreased from 0.17 nM (control) to 0.07 nM after GnX, suggesting an increased GnRH responsiveness of the gonadotroph population (145). Different sizes of gonadotrophs are present in intact mice pituitary gland, most gonadotrophs (97%) were smaller than 60 μm^2^ with a mean of 31.3 ± 0.6 μm^2^ in area and even if large interindividual variation on the peak amplitude of Ca^2+^ transients (Max DF) was seen, no matter the size of the cell, they generated intracellular Ca^2+^ signals smaller than 40 fluorescence arbitrary units (a.u.) poorly correlated with the cell size (Figure 4). By contrast it is reported that 15-day castrated male mouse pituitary gonadotrophs, whose size increase to a mean of 54.4 ± 1.24 μm^2^ and 26% of cells larger than 60 μm^2^ present less variation on the Ca^2+^ peak amplitude and significantly higher correlation of this with the cell size (i.e., hypertrophied gonadotrophs tended to generate Ca^2+^ signals of greater amplitude) (145) (Figure 4), suggesting that in this condition, Ca^2+^ peak amplitude correlated with cell size, and that hypertrophied gonadotrophs tended to produce stronger GnRH-induced Ca^2+^ signals.

Functional adaptation of the gonadotrophs in the pituitary gland to different external and internal conditions may involucrate not just alterations in cell number, size, and morphology, as it has been considered for many years, recent methodological techniques allowed us to understand that it is a more complicated process that involucrates different aspects at the cellular physiology level but in coordination with the whole tissue environment.

## Conflict of Interest Statement

The authors declare that the research was conducted in the absence of any commercial or financial relationships that could be construed as a potential conflict of interest.
